# Long-term effects of simulated microgravity and/or chronic exposure to low-dose gamma radiation on behavior and blood–brain barrier integrity

**DOI:** 10.1038/npjmgrav.2016.19

**Published:** 2016-06-09

**Authors:** John A Bellone, Peter S Gifford, Nina C Nishiyama, Richard E Hartman, Xiao Wen Mao

**Affiliations:** 1 Department of Psychology, Loma Linda University, Loma Linda, CA, USA; 2 Department of Basic Sciences, Loma Linda University, Loma Linda, CA, USA

## Abstract

Astronauts on lengthy voyages will be exposed to an environment of microgravity and ionizing radiation that may have adverse effects on physical abilities, mood, and cognitive functioning. However, little is known about the long-term effects of combined microgravity and low-dose radiation. We exposed mice to gamma radiation using a cobalt-57 plate (0.01 cGy/h for a total dose of 0.04 Gy), hindlimb unloading to simulate microgravity, or a combination of both for 3 weeks. Mice then underwent a behavioral test battery after 1 week, 1 month, 4 months, and 8 months to assess sensorimotor coordination/balance (rotarod), activity levels (open field), learned helplessness/depression-like behavior (tail suspension test), risk-taking (elevated zero maze), and spatial learning/memory (water maze). Aquaporin-4 (AQP4) expression was assessed in the brain after behavioral testing to determine blood–brain barrier (BBB) integrity. Mice that received unloading spent significantly more time in the exposed portions of the elevated zero maze, were hypoactive in the open field, and spent less time struggling on the tail suspension test than mice that did not receive unloading. Mice in the combination group expressed more AQP4 immunoactivity than controls. Elevated zero maze and AQP4 data were correlated. No differences were seen on the water maze or rotarod, and no radiation-only effects were observed. These results suggest that microgravity may lead to changes in exploratory/risk-taking behaviors in the absence of other sensorimotor or cognitive deficits and that combined microgravity and a chronic, low dose of gamma radiation may lead to BBB dysfunction.

## Introduction

Several factors contribute to the health risk faced by astronauts on lengthy voyages outside Earth’s atmosphere and magnetosphere. Among these, exposure to ionizing radiation has been of particular concern not only regarding its effects on physical abilities, but also cognitive functioning. High charge, high energy particle irradiation has been studied most extensively, and has been shown to result in behavioral deficits.^1,2^ Proton particle irradiation has also been linked to deleterious behavioral outcomes.^3,4^ Despite evidence showing impaired functioning following exposure to particle radiation, relatively few studies have assessed the effects of other types of radiation on behavioral endpoints.

Little has been published on the behavioral effects of gamma radiation, especially at low-dose exposure,^5^ and the data from existing literature with rodent models have been mixed. One study, assessing ratio and interval operant responding after gamma (cobalt-60) irradiation, found reduced performance at doses of 4.5–9 Gy, but not at 2.25 Gy.^6^ However, there was a complete recovery by 6 weeks post-exposure in all groups. Another study found no differences in fixed ratio escape performance 6 weeks after 4.5 Gy irradiation, but did find reduced performance at 7.5 Gy for the first 4 weeks followed by a return to pre-irradiation behavior at 6 weeks.^7^

Behaviors other than operant response performance have also been characterized in rodents following gamma radiation. For example, reduced inhibitory control was observed after 5 Gy exposure up to 4 months post-irradiation.^8^ Open field activity, when assessed in aged rats, was altered as a result of 1 Gy *in utero* irradiation, although this was not seen at 0.5 Gy.^9^ Another group showed that *in utero* exposure to 0.5 Gy gamma radiation impaired locomotion, anxiolytic activity, and learning and memory, but did not produce effects at 0.25 Gy.^10^ Overall, the existing gamma radiation literature highlights the relevance of dose and timeline when assessing behavioral effects, with higher doses resulting in greater pathology and a trend toward recovery over a short period of time.

Microgravity has also been identified as a significant risk factor for numerous physical and behavioral abnormalities. It is associated with multiple types of skeletal muscle change and atrophy,^11^ dysregulated gene expression that leads to altered protein synthesis and hemodynamics,^12^ oxidative stress,^13^ altered metabolism,^14^ increased fatigability,^15^ and vestibular issues.^16^ In addition, aquaporin-4 (AQP4), a water channel protein involved in regulating water balance in the brain,^17,18^ has been shown to be altered following hindlimb unloading,^19,20^ a well-established Earth-bound model of microgravity that involves suspending a rodents’ hindlimbs off the ground for an extended period of time (see Materials and Methods for details). It has been suggested^19^ that the increase in AQP4, which is prevalent in the neuromuscular system,^21^ is a response to compensatory mechanisms initiated by muscle atrophy from extended periods of unloading.

Relatively little has been documented regarding cognitive functioning after extended exposure to microgravity, and findings have been inconsistent. Neuron morphology and neuronal networks in culture have shown changes induced by simulated microgravity using a random positioning machine.^22^ Certain genes^12^ and proteins^23^ implicated in learning/memory can also be altered following microgravity, and the resulting changes in cerebral perfusion and ion concentrations^24^ have been associated with cognitive decrements.^25^ In line with this, one study showed decreased accuracy and increased reaction time on an associative memory test.^26^ In contrast, spatial learning/memory was shown to be preserved following spaceflight in rats.^27^ Humans were also found to have intact memory ability both during and after spaceflight.^28–30^ In addition, no changes were found following head-down bed rest, a method of simulating microgravity in Earth-based human studies.^31^ Further data is clearly needed regarding this important topic.

Although radiation exposure and microgravity have been studied separately, data regarding their combined effect is limited. The purpose of this experiment was to study the long-term impact of the separate or combined effects of chronic low-dose/low-dose-rate (LDR) gamma radiation and simulated microgravity on a wide range of behaviors and blood–brain barrier (BBB) integrity. To our knowledge, this is the first study assessing the combined effect of irradiation and microgravity on these endpoints in rodents.

## Results

One mouse died in both the irradiation and combination groups due to weight loss (final *n*=5 for those groups). Mean animal mass for each group prior to exposure to radiation and/or unloading was as follows: control=29.9±0.5 g, radiation only=30.1±0.7 g, unloading only=30.8±1.1 g, and combination=29.4±0.8 g. At sacrifice (9 months post-exposure), masses were as follows: control=38.1±1.1 g, radiation only=37.5±0.9 g, unloading only=40.4±0.9 g, and combination=37.9±1.3 g. There were no significant group differences in pre-exposure mass compared with mass at time of sacrifice.

### Behavior

Mice were tested in the water maze, elevated zero maze, rotarod, and open field at four time points (1 week, 1 month, 4 months, and 8 months post-exposure). They were also tested in the tail suspension test at 4 months post-exposure.

**Figure 1:**
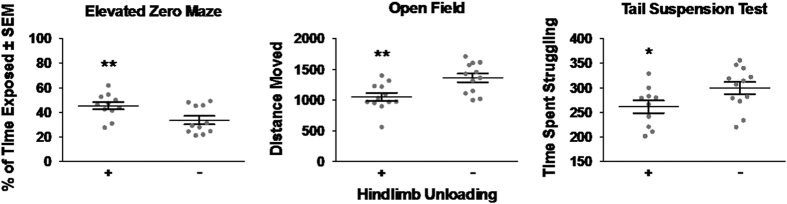
Behavioral data for the elevated zero maze, open field, and tail suspension tests. Mice that received hindlimb unloading spent significantly more time in the exposed portion of the elevated zero maze, were hypoactive during the first exposure to the open field test, and spent less time struggling on the tail suspension test than mice that were not unloaded. *P<0.05. **P<0.01.

A main effect of hindlimb unloading was observed on the elevated zero maze (*F*_1,18_=8.45, *P*<0.01; Figure 1), where the groups that received unloading (i.e., both unloading-only and combination groups) spent a significantly larger percentage of the trial in the exposed portion of the test than mice that did not receive unloading (i.e., radiation-only and control groups). Unloaded mice were also hypoactive during the first 3 min of the open field test compared with mice that were not unloaded (*F*_1,20_=10.73, *P*<0.01; Figure 1). In addition, unloaded mice spent less time struggling on the tail suspension test (*F*_1,18_=4.44, *P*<0.05; Figure 1). LDR colbalt-57 irradiation, simulated microgravity, and their combination had no effect on any parameter of the water maze or rotarod, and radiation did not have an effect on any behavioral endpoint.

### Immunohistochemistry

**Figure 2:**
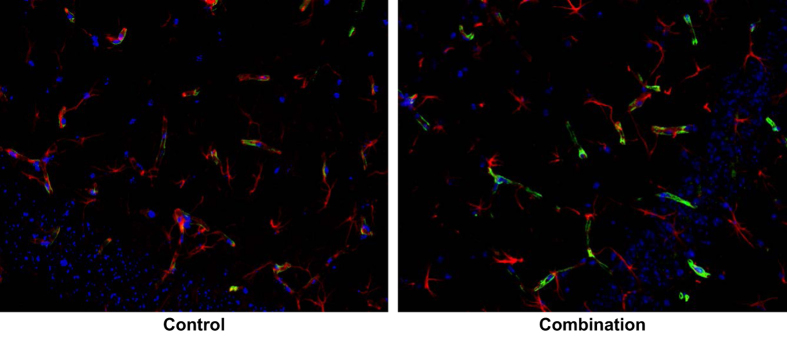
GFAP and AQP4 staining in the brain. Representative micrographs of brain sections were evaluated for astrocytes by immunostaining with anti-4-hydroxynonenal (GFAP) and AQP4 antibodies on samples collected at 9 months after exposure. AQP4-positive staining is identified by green fluorescence, GFAP with red, and the cell nuclei with blue (DAPI). Increased AQP4 staining was seen in the combination group compared with controls. Magnification is x400. DAPI, 4ʹ, 6-diaminodino-2-phenylindole.

**Figure 3:**
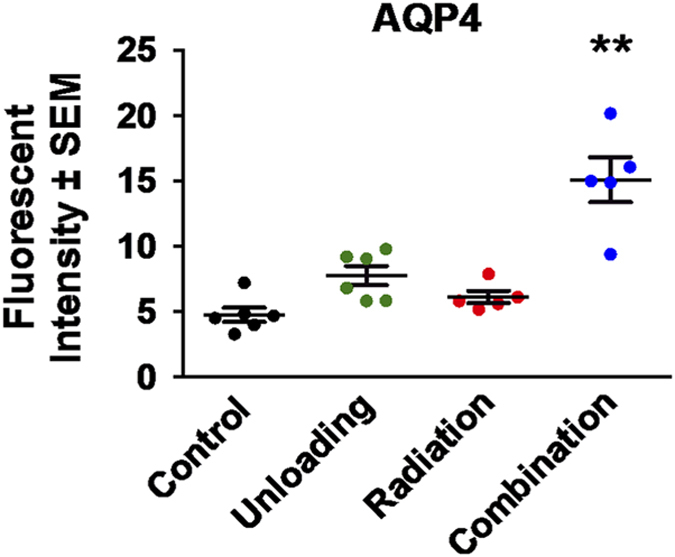
Immunoreactivity of AQP4 staining in the brain at 9 months. The averages of fluorescent intensity for AQP4 activity were measured and calculated using ImageJ. Fluorescence was averaged across five to six mice per group. The combination group showed significantly higher expression than all other groups at 9 months. **P<0.01.

**Figure 4:**
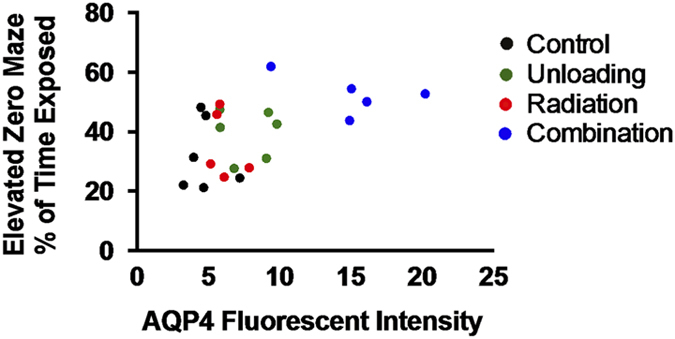
Correlation between percentage of time spent in the exposed portion of the elevated zero maze and AQP4 staining in the brain (*r*=0.50, *P*<0.02). The data were positively correlated, suggesting that BBB compromise is associated with risk-taking behavior.

Two astrocyte markers were used to examine the integrity and function of the BBB: glial fibrillary acidic protein (GFAP) and AQP4. GFAP is present in the cell bodies and large processes of astrocytes. It is an intermediate filament protein involved in the maintenance of the BBB, and is a marker of reactive astrogliosis.^32^ AQP4 is a water channel protein concentrated at the luminal surfaces of astrocyte end-feet, which clearly outline the vascular bed to which they adhere. Increased brain AQP4 staining was seen (Figure 2) after combined irradiation and unloading compared with controls. Main effects of both radiation (*F*_1,18_=20.98, *P*<0.001) and unloading (*F*_1,18_=39.70, *P*<0.0001) were observed. Our data also show significant interactions between radiation and unloading (*F*_1,18_=9.88, *P*<0.01), suggesting that unloading significantly augmented the response to radiation. Tukey’s honestly significant difference *post hoc* comparison showed that the combination group significantly differed from the other three groups (*P*<0.001). As shown in Figure 3, the fluorescent intensity reflecting endogenous levels of AQP4 in the brain was significantly increased in the combination group compared with all other groups at 9 months (*P*<0.01). Higher levels of AQP4 expression were associated with increased time spent in the exposed portion of the elevated zero maze (*r*=0.50, *P*<0.02; Figure 4), suggesting that BBB compromise is associated with risk-taking behavior. There was no notable difference in the pattern of GFAP staining.

## Discussion

In the present study, we exposed 18 female C57Bl/6 mice either to 0.04 Gy LDR cobalt-57 gamma radiation, simulated microgravity (hindlimb unloading), or a combination of irradiation and unloading and measured the effects on behavior up to 8 months, and BBB integrity (by way of GFAP and AQP4 expression) at 9 months, after exposure.

### Behavior

Mice exposed to hindlimb unloading displayed behaviors suggesting abnormal exploration and/or high risk-taking behavior in the elevated zero maze. They were also initially hypoactive in the open field compared with mice that did not receive unloading. Furthermore, the unloaded group spent less time struggling in the tail suspension test, which is likely a sign of habituation to being suspended (due to their prior exposure) rather than suggestive of depression-like behavior. No differences were found in spatial learning/memory (water maze) or sensorimotor coordination (rotarod). Overall, the behavioral data suggest that exposure to simulated microgravity may affect activity level/exploration and increase risk-taking behaviors.

The lack of gamma radiation effects on behavioral measures is not surprising given the aforementioned literature, suggesting that behavioral disturbances primarily manifest following exposure to higher doses^6–8^ than are likely to be encountered in the space environment.^5^ Regarding exposure to microgravity, others have also found no differences in learning and memory. For example, spatial learning and memory was preserved following a 16-day spaceflight in young rats.^27^ The same study found subtle, fleeting differences in exploration patterns. Human studies, both during spaceflight and Earth-bound head-down bed rest models, also did not find substantial differences.^28,29,31^ However, increased risk-taking and exploratory behavior was observed following unloading. Interestingly, changes in risk-taking behavior have been seen in a human head tilt model of microgravity.^33^

Although astronauts occasionally experience mood changes, such as depressive symptoms, while on space missions, there are a variety of factors at play and the unique contribution of microgravity is unknown.^34^ One study demonstrated that spaceflight can modulate certain neuronal mechanisms associated with mood, such as serotonin receptors, dopamine binding, and sodium–potassium pump activity, but indicated that other systems may be spared.^35^ Increased scores on depression inventories and decreases in subjective mood state after head-down bed rest were also found in some studies,^36,37^ but not in others.^31,38^ Bed rest alone can alter mood,^39^ so it is unclear whether effects on mood are due to other factors (e.g., inactivity). We observed subtle unloading-induced changes on the tail suspension test. Although this test is typically viewed as a measure of learned helplessness/depressive behavior in mice, the changes are likely an artifact of the suspension method used to simulate microgravity. Further research is needed to clarify the role of microgravity on emotional functioning.

### BBB integrity

The BBB acts as a diffusion barrier to prevent the inflow of most compounds from the bloodstream to the brain and maintains brain homeostasis.^40^ However, the BBB is more than just a physical barrier; it plays a fundamental role in regulating the movement of substances between the vascular supply of the brain and central nervous system.^41^ Radiation has been documented to cause acute and long-term BBB damage and dysfunction.^42^ BBB dysfunction can compromise immunologically privileged sites and lead to neuroinflammation, as well as modify the progression of neurodegenerative conditions.^43^ Given the high concentration of AQP4 at astrocyte perivascular end-feet and its role in BBB function,^44^ this water channel can act as a marker of BBB integrity. When the BBB is compromised, AQP4 levels tend to increase and are associated with brain edema.^18,45^ The present study found that AQP4 was upregulated as a result of combined exposure to irradiation and unloading, suggesting a disturbance in BBB integrity and edema. Although the AQP4 findings were seen in concert with subtle behavioral changes, the role of BBB dysfunction as a significant pathological factor in the development of behavior disturbance requires further investigation.

## Materials and methods

### Animals

We purchased 6-month-old C57BL/6 female mice (*n*=6 per group), each weighing between 30 and 35 g, from the Jackson Laboratory (Bar Harbor, ME, USA). Sample size was chosen based on our previous studies involving radiation exposure that indicated that sample sizes of five to six per group are adequate to determine differences on our parameters. Animals were maintained under a constant temperature of 68°F with a 12-h day/night cycle. Chow and hydrogel were available ad libitum. After a weeklong acclimatization period, we pre-tested mice using the cued water maze (see below for description) and randomly assigned them to the following performance-matched groups: (1) control, (2) LDR cobalt-57 gamma radiation, (3) hindlimb unloading, or (4) combined unloading and irradiation. The investigators were blinded to the group allocation during behavioral testing and evaluation of immunoactivity. Mice were individually housed during the hindlimb unloading and irradiation period, as well as during each behavioral testing period, but were otherwise housed three per cage. The study was approved by the Institutional Animal Care and Use Committee (IACUC) of Loma Linda University and followed the recommendations of the Guide for the Care and Use of Laboratory Animals of the National Institutes of Health.

### Timeline

We behaviorally tested the mice at four time points: 1 week, 1 month, 4 months, and 8 months after exposure to irradiation and/or unloading. The tail suspension test was only administered at the 4-month time point. The other tests were administered at all four time points. Immunohistochemical analyses of fixed left brain hemispheres were conducted at 9 months after irradiation.

### Hindlimb unloading

Hindlimb unloading by tail suspension is a widely accepted animal model of microgravity that simulates the body fluid changes and mechanical unloading encountered in space-like environments.^46^ For suspension, the tail was inserted into a harness affixed to a guide wire running the length of the top of the cage. The height was adjusted to ensure each animal had its hindlimbs elevated and head tilted 35–40°, with forelimbs free for locomotion and grooming. A grid panel cage floor allowed for animal and food waste to fall through the cage. Control and radiation-only groups were not tail-suspended. Mice were maintained one per cage while they were tail-suspended and irradiated.

### Irradiation

We placed cobalt-57 plates (185 MBq activity; GPI, Stoughton, WI, USA) 7 cm below the cages (1 plate/2 cages) of mice in the radiation and combination groups. A total dose of 0.04 Gy was delivered at an average rate of 0.01 cGy/h over a 21-day period (uniformity of dose was ±5%).

### Behavioral testing

#### Water maze

The water maze is a measure of spatial learning and memory. It consisted of a metal tub (110 cm diameter) filled with water made opaque using white, non-toxic paint. A circular platform (11 cm diameter) onto which mice could step to escape the water was located in one of four quadrants. Each mouse was given 60 s to find the platform. The water maze was comprised of (1) a cued test (day 1), where the escape platform was visible just above the water’s surface (~1 cm) and a stick protruded straight out of the platform to make its location more discernable, (2) two spatial tests (days 2 and 3), where the escape platform was submerged just below (~2 cm) the water’s surface and mice had to rely on spatial cues from around the room to find the platform, and (3) two probe tests (days 2 and 3), where the platform was removed from the pool and mice were allowed to search freely for 60 s 1 h after the completion of the last trial for each spatial test. Ten trials were given in blocks of five each day (two trials per block) during the cued and spatial tests. The platform remained in the same location throughout all 10 trials on the first spatial day (Spatial 1), but changed to a different quadrant for all 10 trials on the second spatial day (Spatial 2). The platform locations and release points changed for each time point. See the study by Bellone *et al.*^3^ for a more detailed methodological description.

#### Elevated zero maze

Although often referred to as a test of anxiety-related behavior,^47^ the elevated zero maze (similar to the plus maze) also assesses risk-taking/exploratory behavior.^48^ The test consisted of a circular track (100-cm outer diameter, 10-cm wide) with two exposed quadrants and two quadrants partially enclosed by walls (35-cm high). Halogen lights directly illuminated the exposed areas of the maze. Mice were placed in the center of one of the exposed areas at the start of the test, and were given 5 min to explore the maze. The percentage of time a mouse spent in the open, exposed space was measured. More time spent in the open areas correlates with increased risk-taking/exploratory behavior.

#### Rotarod

The rotarod consisted of a rotating bar, and mice had to continually move forward to avoid falling. There were three components to this test, with two trials per part: (1) a constant speed of 5 r.p.m., (2) a slow acceleration that started at 5 r.p.m. and increased by 2 r.p.m. every 5 s, and (3) a fast acceleration that started at 5 r.p.m. and increased by 2 r.p.m. every 3 s. Latency to fall was measured. Increased fall latency correlates with better sensorimotor coordination and balance.^49^

#### Open field

The open field test measures activity level and exploratory behavior. Mice were placed alone in a large rectangular box and allowed to explore for 30 min. Walking path was recorded by a computerized tracking system (Noldus Ethovision, Wageningen, The Netherlands) that uses an overhead camera to quantify total distance moved. A novel object was placed in the center of the box in the middle of the trial (15 min) at the 1-, 4-, and 8-month time points to assess whether there was a change in activity level between pre- and post-object placement.

#### Tail suspension test

The tail suspension test involved suspending mice by their tails (via a piece of tape) so that all four limbs were unable to touch the floor. We recorded the amount of time a mouse spent struggling for a 6-min period. Less time spent struggling correlated with learned helplessness/depression-like behavior.^50^

### Immunohistochemistry for AQP4 and GFAP double-labeling

The brain was removed immediately after killing. The left hemispheres were fixed in 4% paraformaldehyde in phosphate-buffered saline (PBS) for immunohistochemistry. Three tissue sections from each brain were selected at 1-mm intervals so as to cover the entire brain region. We washed 20 μm free-floating coronal sections several times in PBS to remove the cryoprotectant storage solution and then blocked in 1% bovine serum albumin (BSA) in PBS for 1.5 h at room temperature. Sections were then incubated overnight (18–21 h) at 4 °C with primary antibodies polyclonal rabbit anti-AQP4 (H-80; 1:500, Santa Cruz Biotechnology, Dallas, TX, USA) and monoclonal mouse anti-GFAP clone GA5 (1:1,000, Millipore, Billerica, MA, USA) in 0.25% BSA, and 0.25% Triton X-100 in PBS. Sections were washed three times in PBS and further treated with secondary antibodies Alexa Fluor 488 goat anti-rabbit IgG and Alexa Fluor 568 Goat anti-mouse IgG (1:1,000, Life Technologies, Pittsburgh, PA, USA). Cell nuclei were counterstained with 4ʹ, 6-diaminodino-2-phenylindole (DAPI, 1 μg/ml, Invitrogen, Carlsbad, CA, USA) and sections were mounted and coverslipped with Vectashield Hard-Set Mounting Medium (Vector Laboratories, Burlingame, CA, USA).

#### Quantification of immunostaining

To determine AQP4 immunoreactivity, fluorescence intensity was measured on 10 randomly selected fields on each section and calculated using ImageJ counting plugin 1.41 software (National Institutes of Health, Bethesda, MD, USA; http://rsbweb.nih.gov/ij/). Once the green channel was separated from the red and blue channels in an image, fluorescence intensities for AQP4 (green channel) from the areas of interest were measured using the integral/density feature in ImageJ. Fluorescence intensities were normalized with respect to controls, and data were extracted and averaged within the group.

### Statistical analysis

We used multi-factorial analysis of variance (ANOVA) with two factors (radiation and unloading) and repeated measures (time point and/or trial, depending on the behavioral test) to assess main effects in the water maze, rotarod, and open field (with object placement). We used two-way ANOVA for the tail suspension test, open field (without object placement), and immunohistochemical data, since there was only one time point for those measures. ANOVA was followed by Tukey’s honestly significant difference *post hoc* multiple-comparison test when appropriate. α-level was set at 0.05 for all tests of statistical significance. Data represent mean±s.e.m.
